# Enhanced cortical bone expansion in *Lgals3*-deficient mice during aging

**DOI:** 10.1038/s41413-017-0003-6

**Published:** 2018-03-26

**Authors:** Kevin A. Maupin, Kevin Weaver, Alexis Bergsma, Cheryl Christie, Zhendong A. Zhong, Tao Yang, Bart O. Williams

**Affiliations:** Center for Cancer and Cell Biology, Program for Skeletal Disease and Tumor Microenvironment, Van Andel Research Institute, Grand Rapids, MI USA

## Abstract

Imbalances between bone formation and bone resorption, which can occur due to aging or sex hormone deprivation, result in decreased bone mass and an increased risk of fracture. Previous studies have suggested that the β-galactoside binding lectin, galectin-3, is involved in bone remodeling. We compared bone parameters of mice having null alleles of the galectin-3 gene (*Lgals3*-KO) with those of their wild-type littermates. *Lgals3* deficiency increased cortical bone expansion at 36 weeks (wk) and preserved or enhanced bone mass in both male and female mutant mice. In addition, female *Lgals3*-KO mice were protected from age-related loss of trabecular bone. Histomorphometry and ex vivo primary cell differentiation assays showed increased osteoblastogenesis with little-to-no effect on osteoclastogenesis, suggesting the increased bone mass phenotype is primarily due to increased anabolism. Our study identifies galectin-3 as a negative regulator of bone formation and suggests that disruption of galectin-3 may be useful in preventing bone loss during aging.

## Osteoporosis: A knockout for stronger bones

Researchers have identified a promising new drug target to reduce bone loss during aging, a protein called galectin-3. Bones undergo lifelong remodeling via resorption of old bone and generation of new bone. With aging, the balance tips towards resorption, weakening bones. Galectin-3 was known to be involved in bone remodeling and levels increased with age. Bart Williams and co-workers at the Van Andel Research Institute in Grand Rapids, USA, investigated whether the age-related increase in galectin-3 increased bone loss. Using mice lacking the gene encoding galectin-3, the researchers measured bone mass at different ages. In older mice, bone mass was preserved or even enhanced. Further investigation of bone cells showed the increase was probably due to increased bone formation, rather than decreased bone resorption. The researchers conclude that disrupting galectin-3 may help to prevent aging-related bone loss.

## Introduction

Osteoporosis is a growing health concern in the United States due to an aging population.^1^ During normal bone remodeling, osteoclast precursors from the myeloid lineage are recruited to areas of old or damaged bone primarily by signals from fully mature osteoblasts, known as osteocytes, which sense changes in bone matrix quality or in mechanical stress.^2,3^ Osteoclast precursors fuse and form a mature osteoclast that is capable of removing damaged tissue via acidic and proteolytic digestion, a process known as bone resorption. This process not only clears out the old bone but also liberates various growth factors from the bone matrix (e.g., Tgfβ, Igf1). These growth factors then recruit osteoprogenitors that proliferate and differentiate into osteoblasts that refill the resorption pit with new bone. This cycle of bone resorption and bone formation allows osteoblasts and osteoclasts to regulate each other’s activities, and while these processes are usually balanced^4^, aging typically leads to a disproportionate increase in bone resorption and subsequently to osteoporosis.^5^

Galectin-3 is a chaperone protein which functions in numerous cell processes intracellularly via protein–protein interactions and extracellularly by binding to specific glycans on glycoproteins.^6^ These interactions are important for polarized glycoprotein secretion^7^ and subcellular localization of galectin-3 binding partners.^8^ Galectin-3 serum levels increase during aging in both mice^9^ and humans.^10^ Galectin-3 regulates inflammation^11^ and *Lgals3*-deficient mice are protected against fibrosis^12–14^, suggesting a potential role for galectin-3 in aging related pathologies. Galectin-3 is expressed in bone, but no one has thoroughly addressed whether *Lgals3*-deficient mice have a skeletal phenotype and whether increased galectin-3 levels during aging play a role in age-related bone loss. Importantly, *Lgals3*-deficient mice have normal reproduction and life span^15^ making it unlikely that galectin-3 inhibition would have serious negative health effects.

Galectin-3 is highly expressed by bone cells (osteoblasts, osteoclasts, and chondrocytes).^16,17^ Previous studies have suggested roles for galectin-3 in osteoblast differentiation^18–20^ and in the suppression of osteoclast function^21^ and recruitment.^21,22^ An early study suggested that adult galectin-3-deficient mice (*Lgals3*-KO) had normal bone size as determined by standard X-ray analysis.^23^ However, later studies suggested that there is increased subchondral bone remodeling in *Lgals3*-KO mice^24^ and that *Lgals3*-KO bones have “a higher quantity of trabecular projections in the marrow cavity” of the femoral diaphysis.^25^ We interpreted the observation of increased trabecular projections as meaning that *Lgals3*-KO mice may have increased cancellous bone mass due to either increased bone formation and/or reduced bone resorption. The same group that noted the increased trabecular projections^25^ also suggested that *Lgals3*-KO femurs were more fragile than wild-type femurs when flushed for bone marrow. Therefore, we hypothesized that *Lgals3*-KO mice would have increased bone mass, but with reduced bone strength. Because galectin-3 serum levels increase during aging^9,10^ and initial studies failed to observe a bone phenotype in young adult mice^23^, we assessed *Lgals3*-KO mice at multiple time points during aging [12, 24, 36, and 48 weeks (wk)] to determine if the phenotype was age-dependent.

## Results

### Loss of *Lgals3* protected against age-related loss of trabecular bone

**Fig. 1:**
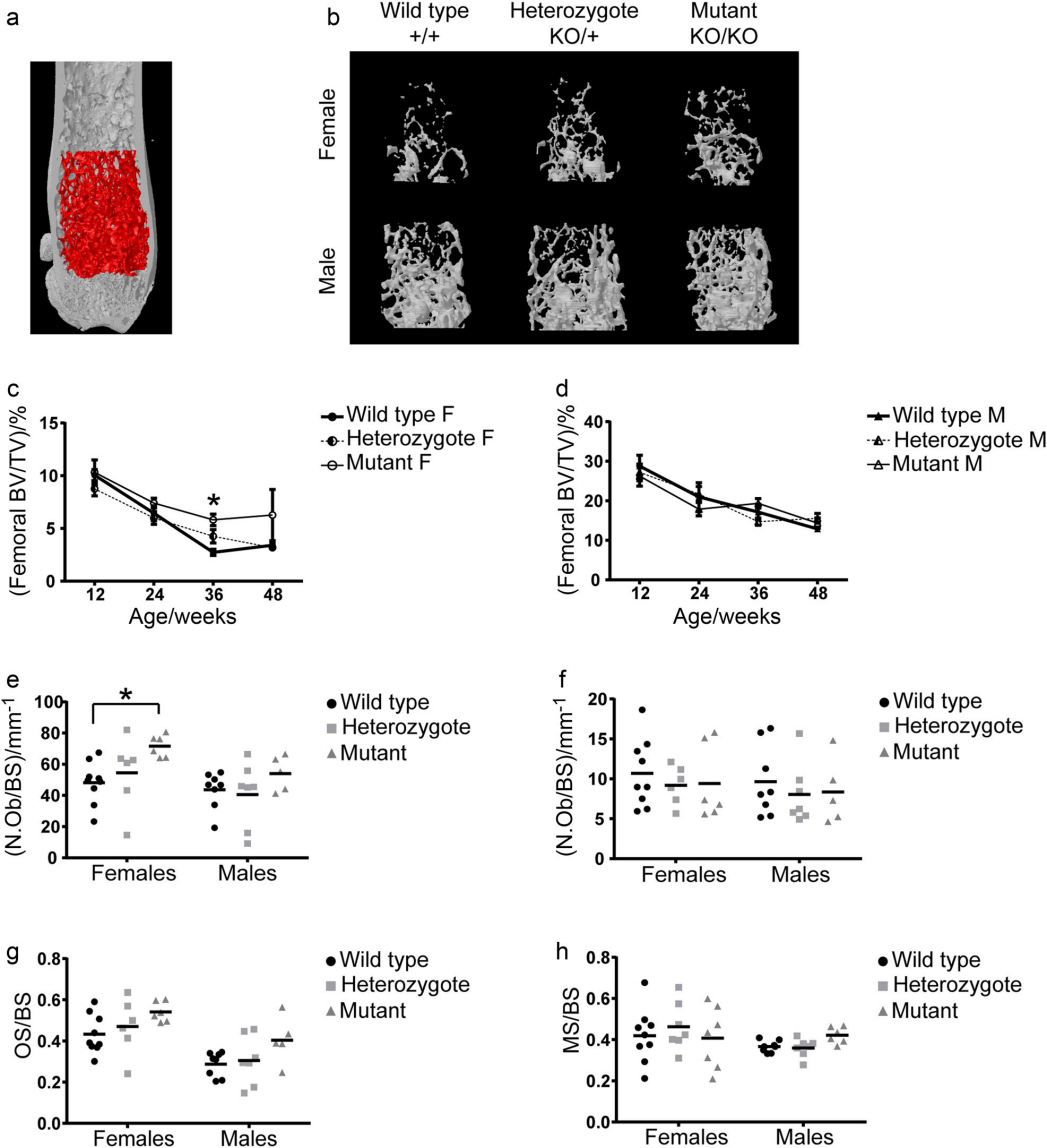
*Lgals3*-deficient females showed protection against age-related trabecular bone loss. **a** Femoral volume of interest is indicated. Representative femoral trabecular bone volumes of 36 wk old female and male mice, as observed by µCT. Average ± S.E.M at indicated time points for bone volume fraction (BV/TV) for female **c** and male **d** mice. Scatterplots from histomorphometry analyses of L3 vertebrae including number of osteoblast per bone surface (N.Ob/BS; **e**), number of osteoclasts per bone surface (N.Oc/BS; **f**), osteoid surface per bone surface (OS/BS; **g**), and mineralized surface per bone surface (MS/BS; **h**). F, Female; M, Male. Asterisks indicate a significant difference, *P < 0.05.

To determine if galectin-3 plays a role in trabecular bone mass, we measured trabecular bone parameters in the distal femur and L3 vertebral body by microcomputed tomography (µCT). The results for femurs and L3 vertebrae are displayed in Supplementary Tables 2 and 3, respectively. While *Lgals3*^KO/KO^ female mice had normal trabecular bone measurements in femurs and L3 vertebrae at 12 wk, by 36 wk they had significantly elevated bone mineral density (BMD) and bone volume fraction (BV/TV) in both the femur (+13.3% and +114.8%, respectively; Fig.1) and the L3 vertebral body (+16.7% and +34.7%, respectively). At 36 wk, the increased BV/TV in *Lgals3*^KO/KO^ femurs was due to significant contributions from increased trabecular thickness (Tb.Th; +14.5%) and trabecular number (Tb.N; +75%), as well as a significant decrease in trabecular spacing (Tb.Sp; −9.5%). The increase in BV/TV in the vertebral body was primarily due to a significant increase in Tb.Th (+12.8%).

**Fig. 2:**
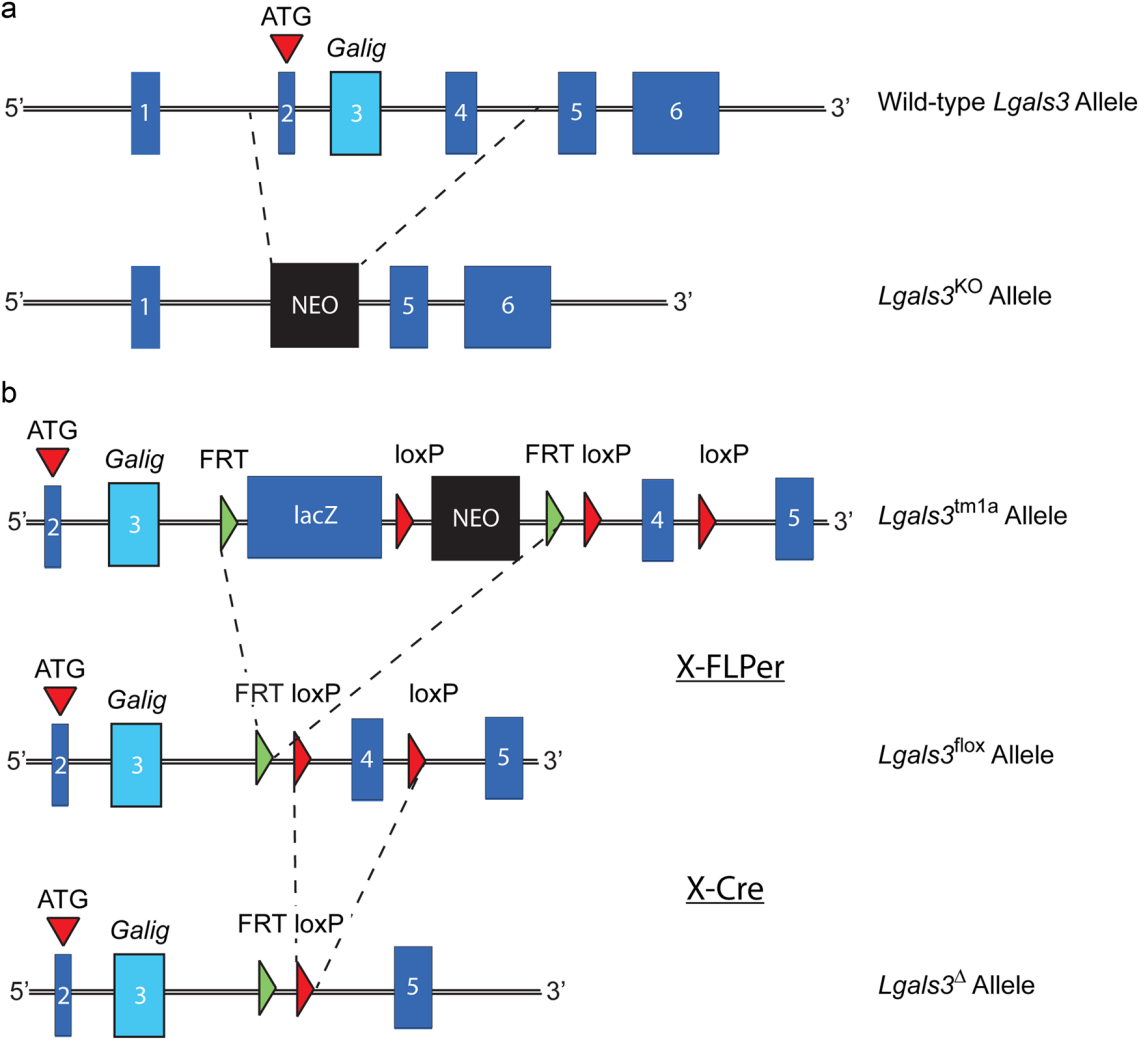
*Lgals3* loss-of-function alleles used in this study. Schematic diagrams showing the genetic perturbations to the wild-type allele including (**a**) the *Lgals3*-KO allele, which was generated by replacing exons 2–4 with a Neo selection cassette and (**b**) the allele generated by EUCOMM that inserted a lacZ reporter and Neo selection cassette flanked by FRT sites and an exon 4 flanked by loxP sites. The lacZ and Neo cassettes were removed following a cross to a mouse expressing the FLP recombinase transgene thereby generating the *Lgals3*-flox allele. Mice with the *Lgals3*-flox allele were then crossed with a mouse expressing the CMV-Cre transgene to obtain mice with a new genomic *Lgals3*-null allele (*Lgals3*-∆).

To determine whether the increased trabecular bone mass that we observed at 36 wk in *Lgals3*-deficient females was restricted to the *Lgals3*-KO allele or the C57BL/6J background, we analyzed bone from mice carrying an independently derived null allele of *Lgals3* (*Lgals3-*∆; Fig.2) maintained on a mixed genetic background. Results from *Lgals3-*∆ are presented in Supplementary Table 4. Trabecular parameters were highly congruent with those of *Lgals3*-KO mice, in that 36-wk *Lgals3*^∆/∆^ females had significantly increased femoral trabecular bone BMD and BV/TV (+40% and +95%, respectively) with a 14% increase in Tb.Th. The L3 BMD was also significantly elevated in *Lgals3*^∆/∆^ females (+23%). Also similar to the *Lgals3*^KO/KO^ mice, we observed no difference in trabecular parameters in *Lgals3*^∆/∆^ males compared to their respective wild-type littermates.

We performed static- and dynamic-histomorphometry on L3 vertebrae of a second cohort of 24-wk *Lgals3*-KO mice (Table 1). Female *Lgals3*^KO/KO^ mice had significantly increased number of osteoblasts per unit of bone surface (N.Ob/BS; +48.6%; Fig.1e). N.Ob/BS was also elevated in male *Lgals3*^KO/KO^ mice (+24%), but this did not reach statistical significance (Fig. 1g). Dynamic histomorphometry revealed no strong relationships between genotype and measurements of trabecular bone formation. There were no changes in measurements of osteoclast number relative to bone surface (N.Oc/BS; Fig.1f).

**Table 1:** Histomorphometry values from L3 vertebrae of wild-type (*Lgals3*^+/+^), heterozygous (*Lgals3*^KO/+^), and mutant (*Lgals3*^KO/KO^) mice at 24 wk^a^

	Females			Males		
Variables compared	Wild type	Heterozygote	Mutant	Wild type	Heterozygote	Mutant
OV/BV	0.06 ± 0.01	0.06 ± 0.01	0.07 ± 0.01	0.04 ± 0.01	0.05 ± 0.01	0.06 ± 0.01
OS/BS	0.43 ± 0.03	0.47 ± 0.06	0.54 ± 0.02	0.29 ± 0.02	0.30 ± 0.05	0.40 ± 0.05
O.Wi/μm	2.93 ± 0.14	3.08 ± 0.17	3.11 ± 0.13	2.80 ± 0.08	2.91 ± 0.20	2.58 ± 0.12
(N.Ob/BS)/mm^-1^	48.22 ± 4.52	54.52 ± 9.43	**71.66** **±** 2.87*	43.61 ± 4.15	40.55 ± 7.82	54.06 ± 5.01
(N.Oc/BS)/mm^-1^	10.70 ± 1.42	9.20 ± 0.98	9.42 ± 1.93	9.64 ± 1.56	8.04 ± 1.44	8.36 ± 1.85
Oc.S/BS	0.29 ± 0.03	0.26 ± 0.04	0.25 ± 0.04	0.20 ± 0.03	0.17 ± 0.03	0.24 ± 0.04
MS/BS	0.42 ± 0.04	0.46 ± 0.04	0.41 ± 0.06	0.37 ± 0.01	0.36 ± 0.02	0.42 ± 0.02
MS/OS	1.03 ± 0.17	1.00 ± 0.16	0.79 ± 0.09	1.35 ± 0.13	1.47 ± 0.22	1.12 ± 0.12
MAR/(μm·d^-1^)	1.01 ± 0.07	1.16 ± 0.13	1.06 ± 0.07	0.72 ± 0.03	0.79 ± 0.04	0.77 ± 0.03
Aj.AR/(μm·d^-1^)	1.06 ± 0.19	1.12 ± 0.16	0.82 ± 0.09	0.98 ± 0.10	1.13 ± 0.13	0.86 ± 0.12
(BFR/BS)/(μm^3^·μm^-2^ per day)	0.43 ± 0.05	0.52 ± 0.05	0.42 ± 0.05	0.25 ± 0.02	0.28 ± 0.02	0.32 ± 0.02
(BFR/BV)/% per day	16.61 ± 1.95	19.16 ± 1.57	15.64 ± 2.05	11.28 ± 1.15	12.94 ± 0.54	14.36 ± 1.01
Omt/d	2.97 ± 0.24	2.75 ± 0.29	3.02 ± 0.25	4.01 ± 0.16	3.58 ± 0.25	3.47 ± 0.31
Mlt/d	3.09 ± 0.62	2.91 ± 0.69	2.40 ± 0.35	5.34 ± 0.42	5.21 ± 0.81	3.80 ± 0.37

OV, osteoid volume; BV, bone volume; BS, bone surface; OS, osteoid surface; O.Wi, osteoid width; N.Ob, number of osteoblasts; N.Oc, number of osteoclasts; Oc.S, osteoclast surface; MS, mineralizing surface; MAR, mineral apposition rate; Aj.AR, osteoid adjusted apposition rate; BFR, bone formation rate; Omt, osteoid maturation time; Mlt, mineralization lag time.

^a^Values are expressed as mean ± S.E.M. (*n* = 6–9). Holm–Sidak post hoc analysis adjusted *P*-values compared to wild type; bold values highlight *P < 0.05.

*Lgals3*-deficient females showed significantly increased growth plate height at 36 wk (*Lgals3*^KO/+^; +25.7%) and 48 wk (*Lgals3*^KO/KO^; +28%), both presented in Table S2. Altogether, the data suggested that the increased trabecular bone measurements at 36 wk in *Lgals3*-deficient females were driven by increased osteoblast numbers and possibly a more active growth plate.

### Loss of *Lgals3* enhanced cortical bone expansion

**Fig. 3:**
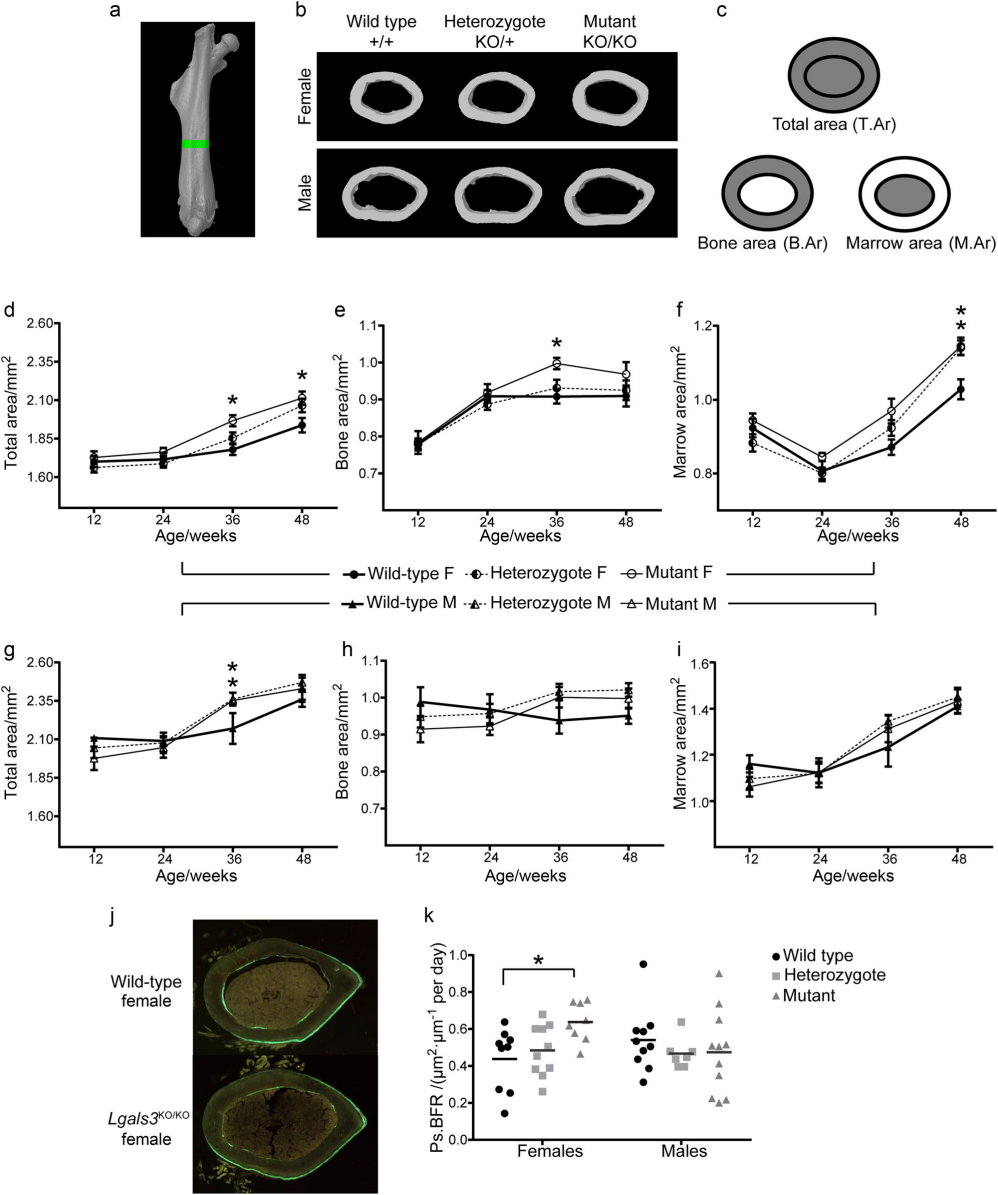
Increased axial expansion of the femur between 24 and 36 wk in *Lgals3*-deficient mice. **a** Femoral region of interest (green). **b** Representative femoral cortical bone sections of 36-wk mice, by µCT. **c** Diagrams corresponding to total area (T.Ar), bone area (B.Ar), and marrow area (M.Ar). Average ± S.E.M. at indicated time points for T.Ar (**d**, **g**), B.Ar (**e**, **h**), and M.Ar (**f**, **i**) for females and males, respectively. **j** Representative calcein labeled sections showing extended region of periosteal double labeling in mutant females. **k** Scatter plot of periosteal bone formation rates. F, Female; M, Male. Asterisks indicate a significant difference, *P < 0.05.

To determine if increased cortical bone expansion could also contribute to the increased B.Ar observed in 36-wk *Lgals3*-deficient animals, we measured changes in cortical bone parameters by µCT. These results are shown in Supplementary Table 5. We observed a significant increase in total area (T.Ar) of both male and female *Lgals3*^KO/KO^ femurs (Fig. 3d, g) starting at 36 wk (+6.5% and +10.7%, respectively). *Lgals3*^KO/KO^ females also had significantly increased T.Ar at 48 wk (+8.8%). Male *Lgals3*^KO/+^ mice also had significantly increased T.Ar at 36 wk (+8.8%). In addition, B.Ar was increased at 36 wk which was significant in *Lgals3*^KO/KO^ females (+9.9%), but failed to reach significance in *Lgals3*^KO/KO^ males (Fig. 3e, h). The smaller cortical bone mass effect observed in *Lgals3*-deficient males may be related to a skeletal growth deficit in young *Lgals3*^KO/+^ (T.Ar; −5.1% and B.Ar; −4.0%) and *Lgals3*^KO/KO^ (T.Ar; −7.9% and B.Ar; −8.1%) mice at 12 wk (Fig. 3g, h), although these results did not reach statistical significance. Young female *Lgals3*-deficient mice had mostly normal or slightly elevated cortical bone parameters at 12 wk, which suggested that galectin-3 may play a role in male bone mass accrual during early development.

We also performed µCT on cortical bone from 36-wk *Lgals3*^∆/∆^ mice and results are shown in Supplementary Table 6. Similar to *Lgals3*^KO/KO^ mice, *Lgals3*^∆/∆^ females had significantly increased B.Ar (+11.6%) and increased T.Ar (+9.5%), but the latter did not achieve statistical significance. Unlike the *Lgals3*^KO/KO^ mice, we did not observe an increase in cortical bone mass in *Lgals3*-∆ males.

Because there was an increase in T.Ar, but no change in cortical thickness in *Lgals3*^KO/KO^ animals, this suggested that the increased B.Ar was primarily to due to increased periosteal bone apposition which was balanced by either a reduction in endosteal bone apposition or increased endosteal bone resorption. Consistent with increased periosteal bone formation driving the increase in T.Ar and B.Ar, dynamic histomorphometry of the femoral midshaft of 24-wk mice (Table 2) showed that *Lgals3*^KO/KO^ females had a significant increase in periosteal bone formation (Ps.BFR; +45.5%; Fig. 3k) that was primarily driven by a significant increase in the periosteal mineral apposition rate (Ps.MAR; +34.4%). There was little change in endocortical bone formation parameters in female *Lgals3*^KO/KO^ mice.

**Table 2:** Cortical dynamic histomorphometry values of wild-type (*Lgals3*^+/+^), heterozygous (*Lgals3*^KO/+^), and mutant (*Lgals3*^KO/KO^) mice at 24 wk^a^

	Females			Males		
Variables compared	Wildtype	Heterozygote	Mutant	Wildtype	Heterozygote	Mutant
Ec.MAR/(μm·d^-1^)	0.69 ± 0.08	0.70 ± 0.06	0.78 ± 0.06	0.66 ± 0.05	0.57 ± 0.05	0.61 ± 0.04
Ec.M.Pm/Ec.Pm	0.65 ± 0.05	0.63 ± 0.05	0.61 ± 0.04	0.70 ± 0.06	0.64 ± 0.05	0.59 ± 0.06
Ec.BFR/(μm^2^·μm^-1^ per day)	0.45 ± 0.07	0.45 ± 0.06	0.49 ± 0.06	0.48 ± 0.07	0.37 ± 0.04	0.36 ± 0.04
Ps.MAR/(μm·d^-1^)	0.61 ± 0.07	0.66 ± 0.04	**0.82** **±** 0.03*	0.81 ± 0.05	0.75 ± 0.04	0.68 ± 0.06
Ps.M.Pm/Ps.Pm	0.71 ± 0.06	0.73 ± 0.05	0.78 ± 0.05	0.66 ± 0.04	0.63 ± 0.03	0.67 ± 0.05
Ps.BFR/(μm^2^·μm^-1^ per day)	0.44 ± 0.06	0.48 ± 0.04	**0.64** **±** 0.04*	0.54 ± 0.06	0.47 ± 0.03	0.47 ± 0.07

Ec, endocortical; Ps, periosteal; MAR, mineral apposition rate; M.Pm/Pm, mineralizing perimeter/total perimeter; BFR, bone formation rate.

^a^Values are expressed as mean ± S.E.M. (*n* = 7–10). Holm–Sidak post-hoc analysis adjusted *P*-values relative to wild type; bold values highlight *P < 0.05.

### Loss of *Lgals3* reduced bone strength and stiffness

**Fig. 4:**
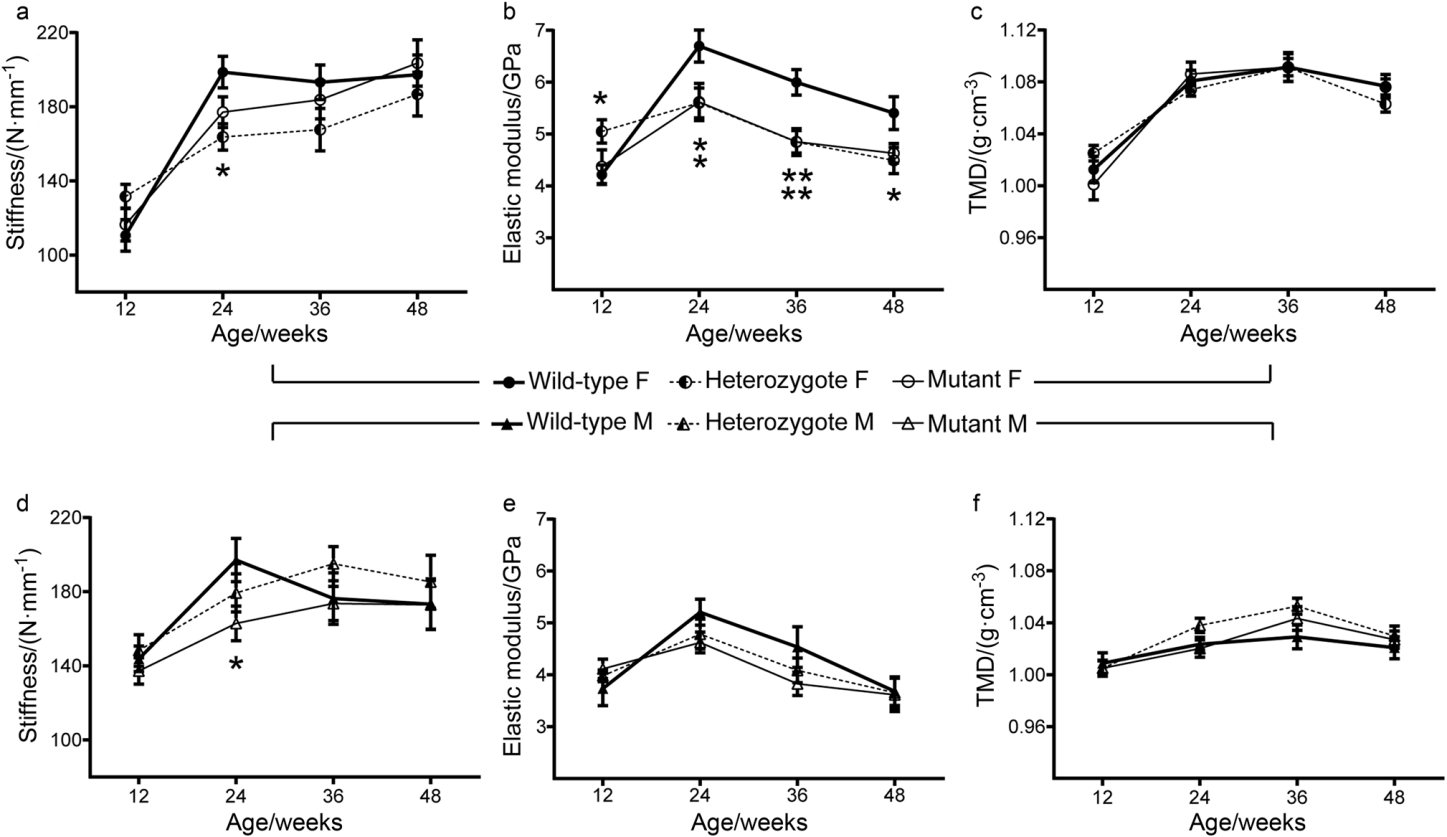
Altered mechanical properties of *Lgals3*-deficient femurs were independent of tissue mineral density. Average ± S.E.M at indicated time points for stiffness (**a**, **d**), elastic modulus (**b**, **e**), and tissue mineral density (**c**, **f**) for female and male mice, respectively. F, Female; M, Male. Asterisks indicate a significant difference, *P < 0.05, **P < 0.01.

The increased T.Ar and B.Ar in *Lgals3*^KO/KO^ animals at 36 wk was accompanied by significantly increased measurements of inertia and normal to slightly elevated tissue mineral density (TMD; Fig. 4c, f), which suggested that femurs from these animals were likely to have improved mechanical strength. To test this, 4-point bending was performed on femurs and full results are shown in Table S5. Despite the improved bone geometry, there was not a corresponding increase in whole-bone strength (max force) or stiffness (Fig. 4a, d) in *Lgals3*-deficient femurs. When max force and stiffness were converted to their tissue level values (max stress and elastic modulus) both *Lgals3*^KO/+^ and *Lgals3*^KO/KO^ female bone tissue withstood significantly less stress at 48 wk (−18.9% and −11.7%) and both had a striking reduction in elastic modulus at 24 wk (−16.4% for both genotypes) and 36 wk (*Lgals3*^KO/+^ : −20% and *Lgals3*^KO/KO^: −18.3%). *Lgals3*^KO/+^ females had significantly reduced elastic modulus at 48 wk (−16.7%), as shown in Fig. 4b.

*Lgals3*-deficient males trended toward a reduction in both max stress (Table S5) and elastic modulus (Fig. 4e) at 24 and 36 wk, and *Lgals3*^KO/KO^ males had a significant reduction in whole bone stiffness at 24 wk (−17.4%; Fig. 4d). These results suggest that while the loss of *Lgals3* led to increased cortical bone formation and normal mineralization, the quality of the deposited bone was relatively lower in aged *Lgals3*-deficient mice, particularly in females.

### Loss of *Lgals3* affected osteoblast, but not osteoclast formation in vitro

**Fig. 5:**
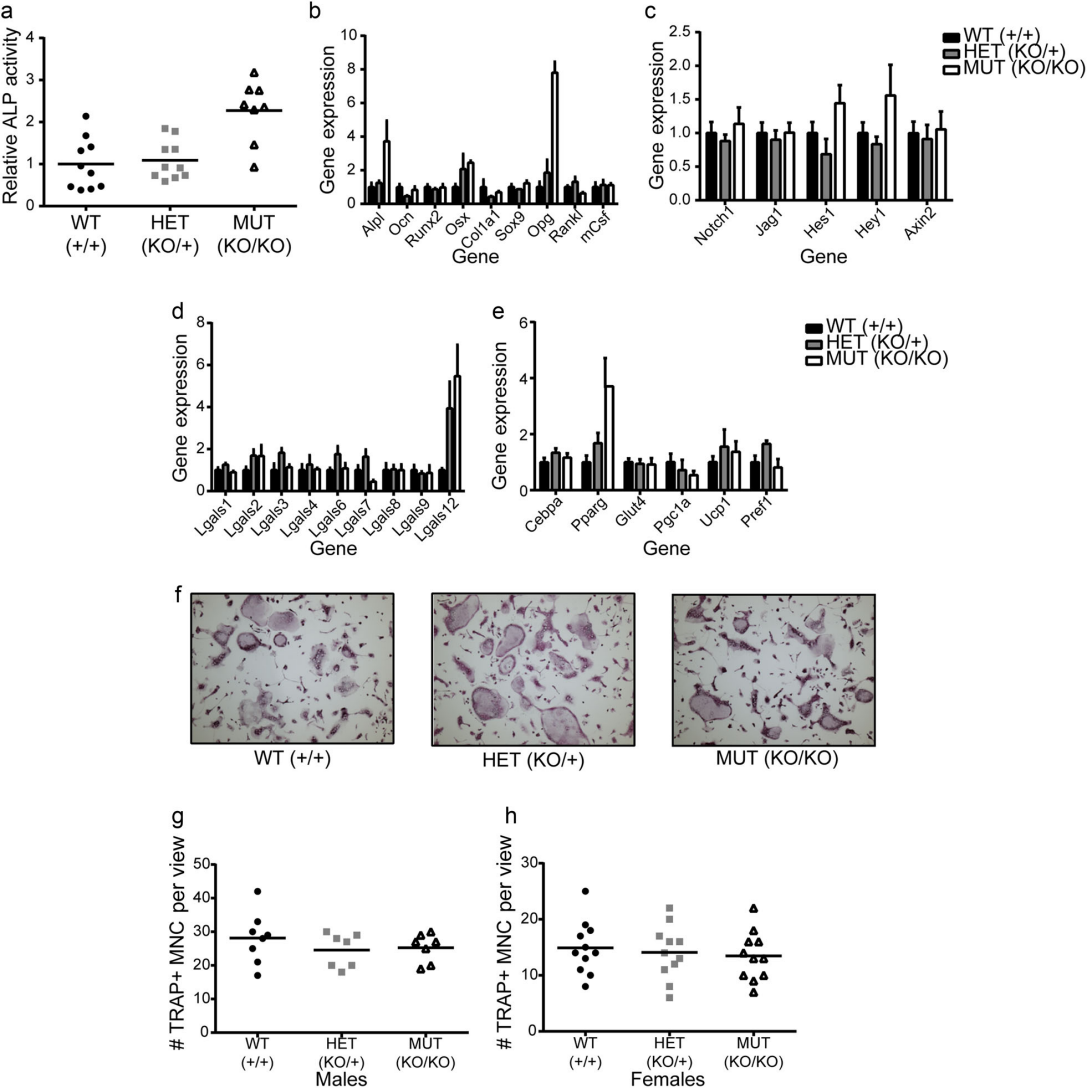
Increased osteoblastogenesis and normal osteoclastogenesis observed following differentiation of *Lgals3*-deficient bone cells in vitro. Calvarial osteoblasts from 2 animals per genotype were cultured for 6 d in osteogenic conditions. **a** Differentiation was analyzed by alkaline phosphatase (ALP) staining (Abs = 405 nm) relative to DNA content (Hoechst 33342; 350 nm, 461 nm) and normalized to wild-type ratios. In identical cultures, gene expression was assessed by qPCR (**b**–**e**). The results represent the mean ± S.E.M. and are representative of duplicate experiments. Bone marrow myeloid cells from 1 animal per sex per genotype were differentiated into osteoclasts for 5 d in osteoclastic conditions. **f** Representative images of osteoclast cultures. Osteoclast counts for male (**g**) and female (**h**) osteoclast differentiation experiments.

We isolated primary calvarial osteoprogenitors from neonatal pups and cultured them with ascorbic acid containing media to stimulate osteoblastogenesis. Calvarial cells isolated from *Lgals3*-KO pups showed enhanced expression of the osteoblast transcription factor, osterix (*Osx*) and alkaline phosphatase on day 6 (Fig. 5b). Congruently, alkaline phosphatase activity was also increased (Fig. 5a). There were no changes in other osteoblast related genes including *Runx2* or osteocalcin (*Ocn*). These results are consistent with increased osteoblast formation from cultures of *Lgals3*^KOKO^ calvarial cells, particularly in the regulation of *Osx* expression.

We also looked at genes involved in osteoblast stimulation of osteoclast differentiation and observed a strong elevation of Opg in *Lgals3*^KOKO^ cells on day 6 (Fig. 5b). Opg suppresses osteoclastogenesis by antagonizing Rankl (receptor activator of NF-kB ligand) signaling^26^ We observed no difference in *Rankl* or macrophage colony stimulating factor (*mCsf*) expression. However, despite the increased transcription of Opg in calvarial osteoblasts in vitro, we actually observed reduced plasma levels of Opg in *Lgals3*-KO mice and alterations to the Opg/Rankl ratio (Table 3), suggesting that there is another factor in vivo that disfavors Opg protein levels in *Lgals3*-KO mice.

**Table 3:** Plasma Opg and Rankl concentrations in wild-type (*Lgals3*^+/+^), heterozygous (*Lgals3*^KO/+^), and mutant (*Lgals3*^KO/KO^) mice^a^

	Females		Males	
Variables compared	24 wk	36 wk	24 wk	36 wk
**Opg/(pg·mL**^**-1**^)				
Wild type	2 662.7 ± 213.0	2 552.5 ± 155.2	2 320.6 ± 137.1	2 751.5 ± 293.8
Heterozygote	2 388.1 ± 189.5	2 216.4 ± 246.1	2 215.3 ± 180.1	**2 153.9** **±** 198.0*
Mutant	2 320.2 ± 70.9	2 205.6 ± 166.5	**1 744.9** **±** 87.4*	**1 900.9** **±** 89.9**
**Rankl/(pg·mL**^**-1**^)				
Wild type	153.4 ± 12.4	152.1 ± 14.3	161.7 ± 16.6	188.4 ± 17.5
Heterozygote	151.7 ± 10.9	175.4 ± 20.7	140.5 ± 8.1	169.9 ± 9.7
Mutant	145.8 ± 10.1	188.9 ± 14.9	124.5 ± 3.9	174.9 ± 7.9
**Opg/Rankl**				
Wild type	17.9 ± 1.7	17.9 ± 2.5	15.2 ± 1.5	14.7 ± 1.1
Heterozygote	16.4 ± 1.9	**13.2** **±** 1.6*	16.3 ± 2.2	13.0 ± 1.6
Mutant	16.4 ± 1.3	**12.3** **±** 1.2*	14.0 ± 0.6	11.1 ± 0.9

Opg, osteoprotegerin; Rankl, receptor activator of nuclear kappa B ligand.

^a^Values are expressed as mean ± S.E.M. (*n* = 6–10); Holm–Sidak post-hoc analysis adjusted *P*-values compared to wild type; bold values highlight *P < 0.05, **P < 0.01.

Due to a recent paper that suggested that galectin-3 negatively regulated osteoblastogenesis by activating Notch1 signaling^19^, we looked at the expression of a set of Notch pathway genes to see if those were conversely downregulated in *Lgals3*-deficient calvarial cells. This did not appear to be the case as *Hes1* and *Hey1* were modestly increased (~ 50% each) in *Lgals3*^KOKO^ cells on day 6 (Fig. 5c). Increased Notch signaling has been observed in T-cells^27^ and dendritic cells of *Lgals3*-KO mice.^28^ This suggests that genomic loss of galectin-3 may lead to increased Notch signaling.

Galectin-3 has been shown to positively regulate β-catenin protein levels in multiple cell types^29–32^, so we were also interested in whether there was evidence of downregulation of Wnt/β-catenin mediated transcription in *Lgals3*-deficient calvarial osteoblasts. However, we observed no difference in the expression of *Axin2* (Fig. 5c), a β-catenin target gene.^33^ Therefore, we do not believe a decrease in Wnt/β-catenin signaling occurs in *Lgals3*-KO osteoblasts, which is consistent with published models showing *reduced* bone mass with reduced β-catenin activity.^34^

We wanted to determine if expression of any other galectin family members might occur in the absence of galectin-3. The most striking change in galectin genes was an ~5.5-fold increase in galectin-12 (*Lgals12*) expression in *Lgals3*^KOKO^ cells after 6 d in osteogenic media (Fig. 5d). Galectin-12 is primarily expressed by adipocytes^35,36^, which prompted us to compare expression of adipocyte related genes from our calvarial cultures. Indeed, we observed a nearly 4-fold increase in *Pparg* transcripts from *Lgals3*^KOKO^ cells (Fig. 5e). However, we did not see significant changes in other adipocyte-related genes from our gene set.

When bone marrow osteoclast progenitors were stimulated to form osteoclasts in response to media containing macrophage colony stimulating factor (mCSF) and receptor activator of nuclear factor κ-B (Rankl), we observed no difference in the formation of multinucleated tartrate resistant acid phosphatase positive cells (TRAP+; Fig. 5f–h). This was consistent with our histomorphometry data where we observed no major changes in osteoclast surface values (Table 1) and suggests that the loss of *Lgals3* does not have a major effect on osteoclast formation.

## Discussion

The identification of genes or proteins that can support gains in bone mass is important for the development of novel therapeutic agents to prevent bone loss due to aging or disease.^37,38^ While there exist efficacious and affordable therapies for preventing bone loss (e.g., sex-hormone replacement^39^ or bisphosphonates^40^), the currently available bone anabolic agents, teriparatide^41^ and abaloparatide^42^, as well as the anti-sclerostin antibodies that may reach market in the near future^43^, are biologics and therefore carry significantly high price tags^40,43^ Their cost combined with their limited duration of effectiveness leaves room for the identification of other anabolic targets, particularly those that are targetable by a less expensive small-molecule inhibitor.^40^ We believe that galectin-3, which has bone anabolic properties, is a suitable target.

Galectin-3 has been purported to associate in various protein complexes in both glycan-dependent (e.g., Itgβ1^44–47^, Muc1^48,49^, and InsR^9^) and glycan-independent (e.g., KRas^50,51^, spliceosome components^52,53^, and Bcl-2^54^) interactions. Most galectin-3 interactions can be disrupted by targeting the glycan binding domain with small molecules^55,56^ known as glycomimetics, a class of drugs whose structures resemble glycans.^57^ This makes it more likely galectin-3 inhibitors could be synthesized in a cost-effective way.

To address the consequence of *Lgals3* deficiency on bone mass during aging, we compared bone parameters of mice carrying 1 or 2 copies of a germline null allele of *Lgals3* (*Lgals3-*KO) to wild-type littermates at 12, 24, 36, and 48 wk by microCT. While bones from *Lgals3*-KO mice were grossly normal, *Lgals3*-KO femurs underwent a period of cortical bone expansion between 24 and 36 wk due to enhanced periosteal bone formation. The cortical bone expansion was stronger in *Lgals3*-KO females, and those mice also showed protection against age-related trabecular bone loss.

We do not yet have a mechanism identified for the age-dependent enhancement of bone mass and cortical expansion in *Lgals3*-KO mice, but we suspect the phenotype may be dependent upon the age-related boost in osteoclast activity that occurs in C57BL/6 mice around the time of the onset of the bone phenotype we observed in *Lgals3*-KO mice.^58^ *Lgals3*-KO mice were recently shown to have increased sensitivity to insulin.^9^ A possible mechanism for the age-dependent bone phenotype could be that *Lgals3*-KO osteoblasts have increased sensitivity to one or more of the growth factors (e.g., Igf1) that are released from the bone matrix during osteoclast bone resorption^4^, leading to super-enhancement of osteoblast activity in high bone turnover states. The study showing the role of galectin-3 in the regulation of insulin sensitivity also showed that serum levels of galectin-3 increase with age.^9^ Therefore, elevations in circulating galectin-3 may inhibit osteoblast function during aging in wild-type mice. Future experiments will be designed to address these potential mechanisms.

Our analyses of *Lgals3*-deficient mice support the concept that targeting galectin-3 might be therapeutically useful for improving bone mass and geometry during aging. However, further mechanistic studies are needed to determine which of the numerous pathways and processes that involve galectin-3 are dysregulated in *Lgals3*-deficient bone cells that may have contributed to the increased bone mass. By better understanding the cellular and molecular mechanisms, compounds could be designed that only target extracellular galectin-3. It may be important to include additional compounds that rescue the decreased material properties of the bone. Nonetheless, the increased bone mass in *Lgals3*-KO mice during aging suggests that the use of galectin-3 inhibitors may prove to be of benefit for skeletal health and longevity.

## Materials and methods

### Animal care

All portions of this study were approved by the Institutional Animal Care and Use Committee at the Van Andel Research Institute (VARI; Grand Rapids, MI, USA). Any animals that showed signs of suffering or distress (e.g., dermatitis, abnormal behavior, injury, etc.) were humanely killed. Mice were monitored every 2–3 d by VARI vivarium staff members trained to identify adverse health events in mice. If mice began showing early signs of an adverse outcome, the cage was marked and monitored daily to determine if the condition advanced or resolved. All mice were housed in climate-controlled conditions (25 °C, 55% humidity, and 12 h of light alternating with 12 h of darkness) and fed standard LabDiet Rodent Chow 5010 (Purina Mills, Gray Summit, MO).

### Germline deletion of *Lgals3* and genotyping

Mice carrying a germline null allele of galectin-3 (*Lgals3*-KO; B6.Cg-*Lgals3*^tm1Poi/J^; 006338) were purchased from the Jackson Laboratory (Bar Harbor, ME). To facilitate studies on a purebred genetic background and produce heterozygous offspring, these mice were crossed with wild-type C57BL/6J, which were purchased from Jackson Laboratory Bar Harbor, ME), and have been maintained in the VARI vivarium. Heterozygotes were set up into breeding pairs to generate litters containing wild type (*Lgals3*^+/+^), heterozygous (*Lgals3*^KO/+^), and homozygous mutant (*Lgals3*^KO/KO^) offspring.

Mice carrying an alternative germline null allele of galectin-3 (*Lgals3*-∆: *Lgals3*^tm1d(EUCOMM)Wtsi^) were generated by first crossing mice carrying a “KO first allele (reporter-tagged insertion with conditional potential)” allele (*Lgals3*^tm1a(EUCOMM)Wtsi^) to mice homozygous for the FLP recombinase transgene (129S4/SvJaeSor-*Gt(ROSA)26Sor*^tm1(FLP1)Dym/J^; 003946) which were purchased from the Jackson Laboratory (Bar Harbor, ME) and maintained at VARI, to remove the LacZ reporter and Neo selection cassette (*Lgals3*^tm1c(EUCOMM)Wtsi^), these mice were then crossed to wild-type C57BL/6J mice to remove the FLPer transgene. Mice from this cross were then crossed to mice homozygous for the Cre-recombinase transgene under control of the CMV enhancer [BALB/c-Tg(CMV-cre)1Cgn/J] to remove exon 4 which was flanked with *loxP* sites. These mice were then crossed to a wild-type C57BL/6J mouse to remove the Cre transgene to obtain *Lgals3*^∆/+^ heterozygous mice. These heterozygotes were set up into breeding pairs to generate litters containing wild type (*Lgals3*^+/+^), heterozygous (*Lgals3*^∆/+^) and homozygous mutant (*Lgals3*^∆/∆^) offspring.

We verified loss of galectin-3 protein in these mice by western blot of lung lysates using a Mac-2/galectin-3 rat monoclonal antibody^59^ (gift from John Wang, MSU, E. Lansing, MI) which recognizes an epitope that is N-terminal of the region encoded by exon 4.^60^ No protein product corresponding to full-length or truncated galectin-3 was detected from lung lysates of *Lgals3*^∆/∆^ mice. The additional benefits of using *Lgals3*^∆/∆^ mice is that they avoid potential transcriptional effects on neighboring genes by a neomycin resistance cassette^61^; also, the genomic deletion is downstream from the galectin-3 internal gene, *Galig*, which utilizes a promoter in intron 2 and encodes a small protein via an alternative open reading frame of exon 3 of *Lgals3*.^62,63^ Figure 2 shows the null alleles utilized in this study.

Genomic DNA was isolated from tail clips at weaning and necropsy by proteinase K digestion and ethanol precipitation. Genotypes were assigned by allele specific PCR. The sequences of primers (synthesized by Integrated DNA Technologies, San Diego, CA) were as follows^1^: The forward primer common to both the *Lgals3*-wild-type and *Lgals3*-KO alleles corresponded to intron 1 of *Lgals3* (5′-GAC TGG AAT TGC CCA TGA AC-3′)^2^, a reverse primer specific for the *Lgals3* wild-type allele corresponded to intron 2 of *Lgals3* (5′-GAG GAG GGT CAA AGG GAA AG -3′), and^3^ a reverse primer specific for the *Lgals3*-KO allele corresponded to the Neo cassette that replaced the region encompassing exons 2–4 (5′-TCG CCT TCT TGA CGA GTT CT-3′). To identify mice carrying the *Lgals3*-∆ allele we used^4^ a forward primer common to both the *Lgals3*-wild-type and *Lgals3*-∆ allele corresponding to intron 3 upstream from the 5′ *loxP* site (5′-GAT CAG AGC AAG AGA TGG GAG-3′)^5^, a reverse primer specific for the *Lgals3*-wild-type allele corresponding to intron 3 downstream from the 5′ *loxP* site (5′-GAC ACA TGA CAT ACA GCA CTG-3′), and a reverse primer to detect the *Lgals3*-∆ allele following Cre-mediated excision of the floxed exon 4 that is located in intron 4, downstream from the 3′ *loxP* site (5′-CTT CAT CTG AAG GCT GCT ATC-3′). The PCR reaction included a hot start at 94 °C for 1.5 min followed by 32 amplification cycles of 94 °C for 30 s, 55 °C for 30 s, 68 °C for 1.5 min + 3 s/cycle. These cycles were followed by 68 °C for 5 min. Then the amplified PCR products were analyzed by 2% agarose gel electrophoresis. For the *Lgals3*-KO reaction (primers 1+2+3), wild-type mice (*Lgals3*^+/+^) yielded a single 220-bp band, homozygous mutant mice (*Lgals3*^KO/KO^) yielded a single 150 bp band, and heterozygotes (*Lgals3*^KO/+^) had both a 220-bp and a 150-bp band. For the *Lgals3*-∆ reaction (primers 4+5+6), wild-type mice (*Lgals3*^+/+^) yielded a single 267-bp band, homozygous mutant mice (*Lgals3*^∆/∆^) yielded a single 501-bp band, and heterozygotes (*Lgals3*^∆/+^) had both a 267-bp and a 501-bp band.

### Plasma isolation and enzyme-linked immunosorbent assays (ELISA)

Immediately following euthanasia, ~0.5 mL of whole blood was collected by heart puncture and transferred to a microcentrifuge tube containing 5 µL of 0.5 mol·L^-1^ ethylenediaminetetraacetic acid (EDTA) pH 8.0. To separate plasma, tubes were centrifuged at 6 000 × *g* for 6 min. The isolated plasma was stored at −80 °C. Samples were thawed on ice and used for ELISA to look at changes in Rankl (receptor activator of nuclear factor kappa B ligand) and osteoprotegerin (Opg) using Quantikine ELISA kits on plasma from 24- and 36-week-old *Lgals3*-KO mice. ELISAs were carried out according to the included protocols (R&D Systems, Minneapolis, MN). Plasma galectin-3 from male and female wild-type and heterozygous mice was measured using a DuoSet ELISA Development System kit (D1197; R&D Systems, Minneapolis, MN). Wells were coated with 100 µL of a 2 μg·mL^-1^ dilution of the galectin-3 capture antibody and only 1 µL of plasma was required per well.

### Micro-computed tomography (µCT)

After euthanasia, right lower limbs and spines were defleshed and fixed in 10% neutral buffered formalin (NBF) for 72 h, rinsed with sterile distilled water, and stored in 70% ethanol at 4 °C. Whole femurs and L3 vertebrae were imaged using a desktop SkyScan 1172 microCT imaging system (SkyScan, Kontich, Germany). Scans for the *Lgals3*-KO aging portion of this study were acquired at 50 kV using a 13.3 μm voxel size. Scans for the *Lgals3*-∆ portion of this study were acquired at 50 kV using a 5.98 μm voxel size. The femoral trabecular volume of interest in the *Lgals3*-KO aging study encompassed regions from 0.25–2.75 mm from the distal growth plate. The femoral trabecular volume of interest in the *Lgals3*-∆ portion of this study encompassed regions from 0.25–1.75 mm from the distal growth plate. For all portions of this study the analyses of trabeculae within the body of L3 vertebrae, a 1.5 mm volume centered on the midpoint was used. For all portions of this study, cortical measurements were obtained from a 0.6 mm segment that was 45% of the distance proximal of the length of the diaphysis from the growth plate (diaphysis length = distance of femoral head-distance of growth plate). Tissue mineral density and bone mineral density values were obtained using a standard regression line generated by converting the attenuation coefficients to mineral density from scans of hydroxyapatite standards with known densities (0.25 and 0.75 g·cm^-3^).

### Mechanical testing

Following euthanasia, left femurs were defleshed, wrapped in phosphate buffered saline (PBS) pH7.2 soaked gauze, and stored at −20 °C. In order to calculate tissue level mechanical parameters we needed to obtain cortical diameter and inertial measurements by µCT. To accomplish this, femurs were thawed at room temperature for 30 min in PBS pH 7.2, analyzed by µCT, scans were acquired at 50 kV using a 13.3 μm voxel size. Cortical regions of interest were determined as previously described for the right femurs. After scanning, the femurs were returned to −20 °C storage. For mechanical testing, the femurs were thawed a second time and allowed to equilibrate to room temperature for 2 h in PBS pH 7.2. Following equilibration, a standard four-point bend testing procedure was performed using a TestResources 570L axial-torsional screw-driven testing system (TestResources, Shakopee, MN) with displacement rate of 0.005 mm·s^-1^. The distances between the lower and upper supports were 7.3 and 3.5 mm, respectively. The supports had radii of curvature of 0.5 mm at each point of contact with the femur. Displacement was applied by the upper supports in the anterior-posterior direction such that the anterior of the femur was in compression and the posterior was in tension. Force and displacement were directly measured from the load cell and crosshead, respectively. Tissue level mechanical parameters (max stress and elastic modulus) were calculated as described for four-point bending^64^ where max stress = (max force*a*c)/(2I_min_) and elastic modulus = stiffness*(a2/(12I_min_))*(3L-4a); where a = the distance between an upper and lower support beam, L is the distance between the lower support beams, I_min_ is the minimum calculated value of inertia, and c is the radius of the bone. I_min_ and c were obtained by µCT.

### Histomorphometry and histology

Fourteen and 4 d before euthanasia, *Lgals3*-KO mice were administered calcein via intraperitoneal injection (20 mg·kg^-1^). Right femurs and spines were dissected at euthanasia and fixed in 10% NBF. Bones were embedded in methyl methacrylate using dibutyl phthalate as the plasticizer (Sigma Aldrich, St. Louis, MO) and sectioned with a tungsten carbide profile D microtome blade (Dorn & Hart, Loxley, AL) to 5 µm thickness using a rotary microtome by the VARI histology core. One section per mouse was analyzed to measure single and double fluorescent labels on cancellous bone in coronal sections of the L3 vertebral body and on cortical bone in transverse sections of the femoral midshaft (Bioquant Osteo 2014, Bioquant Image Analysis Corporation, Nashville, TN). Goldner’s Trichome staining of coronal sections of L3 vertebral bodies was used to get measurements of osteoid, osteoblasts, and osteoclasts. The region of analysis for L3 vertebral bodies encompassed approximately 150–200 µm^2^ of tissue area with boundaries set 250 µm from the growth plates and cortical wall.

Following µCT, right femurs were decalcified in 10% EDTA for 2 weeks prior to paraffin embedding and sectioning. Five-micrometer sagittal sections were cut and fixed to glass microscope slides prior to staining with hematoxylin and eosin. Stains were imaged with a Nikon Eclipse 55i microscope equipped with a Nikon Digital Sight camera (Nikon Instruments, Melville, NY). For measurement of growth plate height, 10 equidistant spans of the growth plate of the distal femur were measured using ImageJ software (US National Institutes of Health, Bethesda, MD, USA) and averaged.

### Primary cell isolation and differentiation

Osteoblast progenitors were isolated from calvariae of newborn *Lgals3*-KO mice by serial digestion with 1.2 mg·mL^-1^ collagenase type I (Worthington Biochemical, Lakewood NJ) in Hanks buffered saline solution (HBSS). Calvariae were placed in 10 mL of collagenase solution for 15 min at 37 °C with shaking at 90 r·min^-1^. The collagenase solution was decanted and replaced with fresh collagenase and the digestion was repeated. This was performed a total of five rounds; only supernatants from first two rounds were discarded. Supernatants 3–5, containing osteoblasts and osteoprogenitors, were pooled. Cells were cultured in alpha-minimum essential medium (α-MEM; Gibco, Life Technologies Corporation, Grand Island, NY) with 10% heat-inactivated fetal bovine serum (FBS; Hyclone, GE Life Sciences, Logan, UT) and 1% penicillin/streptomycin (Gibco, Life Technologies Corporation, Grand Island, NY). Cultures were maintained at 37 °C in a fully humidified atmosphere of 5% CO_2_ in air. After 3 d, primary calvarial osteoblast cultures were allowed to expand and subconfluent cells were passaged into 96-well plates at 10 000 cells per well. After 2 d, the media was changed to osteogenic media (α-MEM containing 10% FBS, 1% penicillin/streptomycin, 50 µg·mL^-1^ ascorbic acid, and 2.16 mg/ml β-glycerophosphate). RNA was collected in TRIzol (Invitrogen, Carlsbad, CA) on day 6 to assess changes in gene expression. Alkaline phosphatase activity was assessed using 1-Step NBT/BCIP solution (Thermo-Fisher Scientific, Waltham, MA) in separate cultures on day 6.

Osteoclast progenitors were isolated from the bone marrow of femurs and tibias of 5-week-old *Lgals3*-KO mice. Marrow was collected by brief centrifugation into microcentrifuge tubes (allowed centrifuge to ramp up to 14 000 × *g* and held for 5 s). Marrow was reconstituted in 2 mL of ammonium-chloride-potassium (ACK) lysis buffer and incubated at room temperature for 10 min to lyse red blood cells. Cell solution was then passed through a 70 µm filter and centrifuged at 1 800 x *g* at room temperature for 5 min to pellet cells. Lysis buffer was removed from the cell pellet by vacuum, followed by resuspension in 10 mL of α-MEM (Gibco, Life Technologies Corporation, Grand Island, NY) with 10% heat-inactivated fetal bovine serum (FBS; Hyclone, GE Life Sciences, Logan, UT), 1% penicillin/streptomycin, and 1% L-glutamine. For simplicity, we will refer to this media cocktail as α-MEM1. The cell suspension was cultured on a 10 mL plate at 37 °C in a fully humidified atmosphere of 5% CO_2_ in air for 24 h to remove adherent cells.

Cells remaining in suspension were counted and plated in a 24-well plate at 200 000 cells in 0.5 mL of α-MEM1 + 20 ng·mL^-1^ macrophage colony stimulating factor (mCSF; R&D Systems, Minneapolis, MN) to expand the macrophage progenitor pool. After 2 d, the cell suspension was carefully removed and transferred to a new well of a 24-well plate for osteoclast differentiation. Osteoclast differentiation was induced by replacing media with fresh α-MEM1 treated with 20 ng·mL^-1^ mCSF and 20 ng·mL^-1^ Receptor activator of nuclear factor kappa B ligand (Rankl; R&D Systems, Minneapolis, MN). Control wells were treated with α-MEM1 treated with mCSF only. Differentiation media was replenished after 3 d with the aforementioned final concentrations of mCSF and Rankl. Differentiation ended on day 5 and cultures were stained for tartrate-resistant acid phosphatase (TRAP) activity using the Acid Phosphatase, Leukocyte (TRAP) Kit (Sigma Aldrich, St. Louis, MO). Osteoclasts were identified as TRAP-positive cells with more than two nuclei and counts were averaged across images selected at random.

### RNA extraction, cDNA synthesis, and qPCR

Total RNA was extracted using Trizol reagent (Invitrogen, Carlsbad, CA) according to the manufacturer's instructions. Then cDNA was synthesized using random hexamers. qPCR was carried out using the StepOnePlus Real Time PCR System (Applied Biosystems, Foster City, CA). Reactions were run in duplicate in two independent experiments. The geometric mean of housekeeping gene β-actin was used as an internal control to normalize the variability in expression levels. Expression data were normalized to the geometric mean of housekeeping gene β-actin and fold changes were calculated using the 2^−ΔΔCT^ method^65^, then normalized to wild type expression levels for each gene. Galectin primer sets were purchased from Origene (Rockville, MD). All other primer sets were synthesized by IDT (San Diego, CA). Primer sequences can be found in Supplementary Table 1.

### Statistical analyses

For the aging study, differences between *Lgals3* wild-type (+/+) and *Lgals3*-deficient (KO/+ and KO/KO) mice were determined using two-way ANOVAs within age groups (sex, genotype). For the galectin-3 plasma ELISA, differences between *Lgals3*-wild-type (+/+) and *Lgals3*-heterozygous (KO/+) mice were determined using two-way ANOVAs within sex (age, genotype). The Holm-Sidak method was used in post hoc analyses to identify significant differences (α = 0.05). All statistics and graphs were generated using GraphPad Prism 6 (GraphPad Software Inc., La Jolla, CA).

## Electronic supplementary material

Supplemental Table 1

Supplemental Table 2

Supplemental Table 3

Supplemental Table 4

Supplmental Table 5

Supplemental Table 6
